# Refocusing neuroscience: moving away from mental categories and towards complex behaviours

**DOI:** 10.1098/rstb.2020.0534

**Published:** 2022-02-14

**Authors:** Luiz Pessoa, Loreta Medina, Ester Desfilis

**Affiliations:** ^1^ Department of Psychology, University of Maryland, College Park, MD 20742, USA; ^2^ Department of Experimental Medicine, Institut de Recerca Biomèdica de Lleida Fundació Dr. Pifarré (IRBLleida), University of Lleida, 25198 Lleida, Spain

**Keywords:** mental functions, structure–function relationship, brain evolution

## Abstract

Mental terms—such as perception, cognition, action, emotion, as well as attention, memory, decision-making—are epistemically sterile. We support our thesis based on extensive comparative neuroanatomy knowledge of the organization of the vertebrate brain. Evolutionary pressures have moulded the central nervous system to promote survival. Careful characterization of the vertebrate brain shows that its architecture supports an enormous amount of communication and integration of signals, especially in birds and mammals. The general architecture supports a degree of ‘computational flexibility’ that enables animals to cope successfully with complex and ever-changing environments. Here, we suggest that the vertebrate neuroarchitecture does not respect the boundaries of standard mental terms, and propose that neuroscience should aim to unravel the dynamic coupling between large-scale brain circuits and complex, naturalistic behaviours.

This article is part of the theme issue ‘Systems neuroscience through the lens of evolutionary theory’.

## 1. Introduction

**Figure 1.**
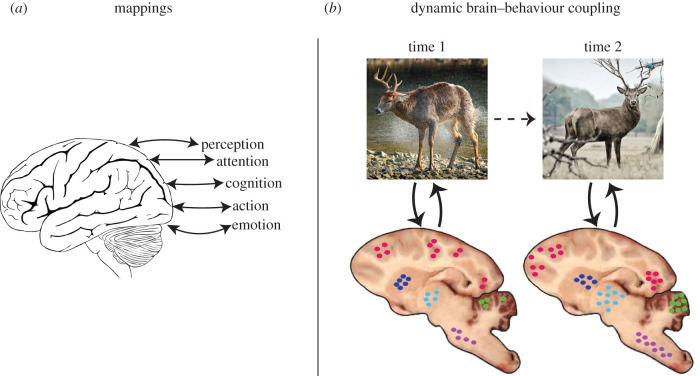
Neuroscience and mental terms. (*a*) One of the central problems in neuroscience is to discover mappings between brain and mental functions. (*b*) Characterizing the coupling between distributed, and dynamic neural circuits, and complex, dynamic behaviours. As behaviours unfold temporally, large-scale circuits spanning the neuroaxis are dynamically recruited to support them. The different colours schematically indicate neuronal populations in different parts of the brain. Although the images at time 1 and time 2 illustrate different ‘states’, one can envisage continuous behavioural and neuronal ‘trajectories’. A central goal of neuroscience should then be to understand the coevolution of the two classes of trajectory. (Online version in colour.)

Open a textbook on the mind and brain, say *Cognitive Neuroscience: The Biology of the Mind* by Gazzaniga *et al*. [1]. Skimming through it, we see chapters on perception, attention, memory, learning and development, language, motor control, executive functions (‘higher’ cognitive functions) and consciousness. Aside from consciousness, which comes at the end of the book as a challenging subject, the other topics sound well defined and even intuitive. A central goal of neuroscience is then to uncover how these mental functions are instantiated in the brain (figure 1*a*).

But where do the above chapter themes come from? Take *attention*, for example [2].^1^ In the West, ideas about attention have been discussed since the Greeks. For example, Aristotle questions whether ‘ … it is possible or not that one should be able to perceive two objects simultaneously in the same individual time?’ This problem is echoed in modern research when Huang & Pashler [3] state that ‘[t]his question about [the] possibility of simultaneous selection of two feature values is very fundamental’. Perhaps closer to intuitive notions of attention, consider what is now called the ‘cocktail party effect’, as formulated a few centuries ago by Stewart [4]): ‘When two persons are speaking to us at once, we can attend to either of them at pleasure. … This power, however, of the mind to attend to either speaker at pleasure, supposes that it is, at one and the same time, conscious of the sensations which both produce’.

The terms above—perception, attention, etc.—have long histories, and make for excellent chapter headings in a textbook. But do they provide reasonable conceptual anchors for neuroscience (see also [5])? Consider *perception*, which involves processing elements of the environment ‘through physical sensation’.^2^ What is the relationship between perception and other mental processes? The classical sequential scheme has long been supplanted by interactive schemes with feedback and other interactions. Thus, perception is not separate from cognition, and not even from action if one adopts ‘active’ frameworks [6–8]. More broadly, perception is used to denote a large number of processes only loosely related. For the experimental psychologist or neuroscientist, the concept is just too vague and lacks sufficient coherence to provide much conceptual utility. Perhaps perception is so basic that the criticism does not apply to other mental terms commonly used in neuroscience (say, ‘attention’). Instead, we argue that the problems with what we will call *standard mental terms* are quite general.

Mental categories are routinely used in a dual fashion to denote both the problem—the phenomenon one aims to explain—and the solution—the mechanism proposed to provide the explanation [9].^3^ More broadly, mental terms are used in a *circular* fashion. For example, ‘emotional processing’ is defined in terms of systems that are purported to be part of the emotional brain, such as the hypothalamus or the amygdala; conversely, a structure that plays an important role in ‘fear’ is considered part of the emotional brain. Although the language used in some instances is not necessarily flawed, such linguistic habits are potent enough to lead investigators astray, and limited knowledge is actually gained by sticking to the traditional terminology.

More forcefully, we propose that mental terms are epistemically sterile. We support our thesis based on extensive comparative neuroanatomy knowledge of the organization of the vertebrate brain. Evolutionary pressures have moulded the central nervous system to promote survival. Careful characterization of the vertebrate brain shows that its architecture supports an enormous amount of communication and integration of signals. The general neuroarchitecture supports a degree of ‘computational flexibility’ that enables animals to cope successfully with complex and ever-changing environments. Here, we suggest that the vertebrate neuroarchitecture does not respect the boundaries of mental terms (see also [10]).

Thus, we propose that neuroscience should seek to unravel the coupling between large-scale circuits spanning the neuroaxis and complex, naturalistic behaviours and critically how the temporal evolution of behaviour is linked to dynamic brain changes (figure 1*b*). Large-scale circuits discussed below include those involving the basal ganglia, amygdala and superior colliculus/optic tectum systems, which illustrate how the vertebrate neuroarchitecture contains a series of spiralling pathways that communicate and integrate signals across different spatial extents (see also [11,12]).

Our critique of a form of ‘unrestrained mentalism’ common in neuroscience does not entail a return to *behaviourism*. However, we do believe that the careful characterization of behaviour is fundamental in neuroscience, and deserves considerably more attention (e.g. [13]), in particular *ethological approaches* focusing on natural behaviours and *comparative approaches* that consider a range of species.

Before proceeding, we stress that our discussion is selective given space restrictions. In particular, we were unable to review multiple lines of research at the interface between psychology and neuroscience that have emphasized ‘natural behaviours’; for example, the work by Panksepp [14], including his work on play behaviours; as well as the work by Berridge *et al*. [15] on how the liking/wanting distinction informs the understanding of behaviours such as feeding, mating and parental care. Additionally, Barrett & Satpute have developed related ideas, for example, proposing a focus on functionally integrated brain systems, and voicing concerns about standard mental domains (e.g. [16]). Regarding the latter, Uttal [17] has forcefully voiced opposition to the notion that ‘cognitive’ processes can be localized in the brain; for more constructive views in the domain of, for example, memory, see Fuster [18].

## 2. Emotion and cognition

To illustrate how the semantic separation of mental terms has helped shape the understanding of their neural basis, let us discuss some of the origins of how emotion and cognition are viewed as segregated in the brain.

In the conclusion of *The Descent of Man*, Darwin wrote in 1871 that ‘the indelible stamp of his lowly origin’ could still be discerned in the human mind, with the implied consequence that it was necessary to suppress the ‘beast within’. This notion was hardly original, of course, and in the West can be traced back to at least ancient Greece. At Darwin's time, with emotion being considered primitive and reason the more advanced faculty, ‘true intelligence’ was viewed as residing in cortical areas, most notably in the frontal lobe, while emotion was viewed as residing in the basement, the lowly brainstem.

The decades following the publication of Darwin's *Origin of Species* (in 1859) were a time of much theorizing not only in biology but in the social sciences, too. Herbert Spencer and others applied key concepts of biological evolutionary theory to social issues, including culture and ethics. *Hierarchy* was at the core of this way of thinking. For the survival of evolved societies, it was necessary to legitimize a hierarchical governing structure, as well as a sense of self-control at the level of the individual [19]. These ideas, in turn, had a deep impact on neurology. Hughlings Jackson, to this day the most influential English neurologist, embraced a hierarchical view of brain organization rooted in a logic of evolution as a process of the gradual accrual of more complex structures atop more primitive ones. Thus, ‘higher’ centres in the cortex exert control on ‘lower’ centres underneath, and any release from this control could make even the most civilized human act more like their primitive ancestors. This stratified scheme was also enshrined in Sigmund Freud's framework of the *id* (the lower level) and the *super-ego* (the higher level). Against this backdrop, it is not surprising that brain scientists would search for the neural basis of emotion in subcortical territories, while viewing ‘rational thinking’ as the province of the cerebral cortex, especially the frontal lobe.

In 1896, the German anatomist Edinger [20] published *The Anatomy of the Central Nervous System of Man and of Vertebrates in General*. The book, which established Edinger's reputation as the founder of comparative neuroanatomy, described the evolution of the forebrain as a *sequence of additions*, each of which established new brain parts that introduced new functions.

Edinger viewed the forebrain as containing an ‘old encephalon’ found in all vertebrates. On the top of the old encephalon, there was the ‘new encephalon’, a sector only more prominent in mammals. In one of the most memorable passages of his treatise, Edinger illustrates his concept by asking the reader to imagine precisely inserting a reptilian brain into that of a marsupial (a ‘simple’ mammal). When he superimposed them, the difference between the two was his ‘new encephalon’. He then ventured that, in the brain of the cat, the old encephalon ‘persists unchanged underneath the very important’ new encephalon. Put differently, the part that was ancestrally present is left unaltered. Based on his coarse analysis of morphological features, his suggestion was reasonable.^4^

But to a substantial degree, his ideas were very much in line with the notion of brain evolution as progress towards the human brain, as in the Aristotelian notion of the *scala naturae* [21]. Given the comprehensive scope of Endinger's analysis across vertebrates, his views had a lasting impact and shaped the course of research well into the 1960s.

More than a century later, knowledge about the brains of vertebrates has expanded enormously. Yet, old thinking dies hard. Antiquated views of brain evolution continue to influence neuroscience, even if implicitly. As an example, consider that most frameworks of brain organization are heavily centred on the cortex. These descriptions view ‘newer’ cortex as controlling subcortical regions, which are assumed to be (relatively) unchanged throughout evolution. Modern research on brain anatomy from a comparative and functional architecture viewpoint indicates, in contrast, that brain evolution is better understood in terms of (i) modification in neuronal populations within the brain's fundamental units (building blocks) and (ii) the reorganization of large-scale connectional systems in which they are engaged, as described below (for a more detailed treatment, see [12]).

Yet, textbooks often present ‘cognitive’ and ‘emotional’ systems as if they were separate entities (this is especially the case in clinically oriented materials). In particular, textbooks still discuss the ‘limbic brain’, a concept that has no stable meaning, and essentially is used as an amorphous synonym for a putative ‘emotional brain’.^5^ What is more, the purported ‘emotion/cognition’ separation continues to generate ideas of wide public appeal, such as the notion of ‘System I/System II’ popularized by Kahneman [22].

## 3. Basic principles of the vertebrate forebrain

The brain of all vertebrates is organized according to a common ‘building plan’, also called Bauplan or morphoplan [23,24], which shares the same basic subdivisions (reviewed in [25]). To unravel the common architecture, it is critical to identify and map the same—technically, *homologous*—brain regions in different vertebrates. Homology refers to relationships between traits that are shared as a result of common ancestry [26]. For example, the human arm and the bird wing are considered homologous as upper limbs because they arose from a corresponding character in the tetrapod common ancestor through descent with modification. Note, however, that they differ greatly in terms of both detailed structure (distal parts related to adaptive features) and function.

Evolutionary divergence can make it difficult to identify homologies. In such cases, the study of embryonic development helps in detecting homologies, as embryos of different species are more similar to one another than adults. This approach has been successfully applied to the comparative study of the organization of the vertebrate nervous system. During development, a series of segments, called ‘neuromeres’, can be identified that form essential building blocks of the central nervous system [24]. Sets of neuromeres form key morphological entities that can be identified during development, and give rise to three major territories in adults: forebrain (prosencephalon), midbrain (mesencephalon) and hindbrain (rhombencephalon). In the forebrain, the *telencephalon* can be subdivided into two major divisions: the *pallium* and the *subpallium*, which we will discuss next. (If the reader is unfamiliar with these terms, as the first approximation they can mnemonically link ‘pallium’ (from the Latin word for woolen cloak or mantle) with the cortex and ‘subpallium’ with the basal ganglia, among other areas in the ventral telencephalon, although this is not strictly correct; see below).

### (a) Pallium

In mammals, the pallium includes the cortex in addition to non-cortical structures, such as the claustrum and the *basolateral complex* of the amygdala—we call the latter the *pallial amygdala*.

At present, there are competing views about the overall plan of the vertebrate forebrain (reviewed by the authors in [23,27]). Controversy is particularly acute regarding the telencephalon, the largest subdivision of the forebrain in amniotes (reptiles, birds and mammals). This part of the brain shows a high degree of divergence, although it shares the same basic divisions in different groups. One view of the vertebrate morphoplan is the *developmental genoarchitecture* hypothesis, which is based on shared expression patterns of highly conserved regulatory genes observed at early embryonic stages [28,29]. When comparative genoarchitecture data are integrated with key morphological landmarks, the embryonic pallium of vertebrates can be subdivided into four [30] or six [31] compartments that are comparable across species. To unify the proposals of four versus six pallial divisions, we refer to them as medial, dorsal, lateral (more precisely, dorsolateral/lateral) and ventral (more precisely, ventral/ventrocaudal) pallial divisions.^6^

According to the developmental genoarchitecture proposal, the medial pallium gives rise to the hippocampal formation [25,34,35]. In mammals, the dorsal pallium gives rise to the isocortex (with six layers), the dorsolateral/lateral pallium produces the claustro-insular region, the orbitofrontal cortex and the perirhinal/lateral entorhinal cortex, while the ventral pallium gives rise to the olfactory cortex and part of the amygdala, the so-called pallial amygdala [30,31,35,36]. Based on data on the development of the amygdala [37], we can also consider that the ventral pallium includes a rostral sector that produces the piriform cortex plus endopirifom nuclei and a distinct caudal sector that produces the pallial amygdala.

### (b) Basal ganglia loops

Across vertebrates, the subpallium is relatively conserved and contains the striatum, pallidum (not to be confused with ‘pallium’), parts of the amygdala (subpallial amygdala) and the bed nucleus of the stria terminalis (BST), among others [35,38,39]. Here, we discuss a central element of the neuroarchitecture of tetrapod vertebrates (amphibians, reptiles, birds and mammals), which involves cortical–subcortical (more generally, pallial–subpallial) forebrain circuits.

Classically linked to movement control and disorders, the basal ganglia are now known to be involved in multiple functions and viewed as essential for sophisticated forms of behavioural control, including learning and regulation of stimulus-driven behaviours, as well as action selection supporting goal-directed behaviours [40–42]. Work focusing on mammals in the 1970s–1980s uncovered the cortical–subcortical connectional architecture of the basal ganglia [43,44]. Nearly the entire cortical sheet projects to the striatum, and whereas striatal territories receiving cortical input do not directly reciprocate their connections, pathways return to cortex via different parts of the thalamus after an additional step in the pallidum. Together, these studies have led to the important concept of cortico-basal ganglia–thalamo-cortical systems, or *basal ganglia loops* in short [43,45].

An important feature of mammalian basal ganglia loops is that they involve both dorsal (caudate-putamen) and ventral (nucleus accumbens) striatal components. Some cortical areas project to the dorsal striatum (for example, motor and somatosensory areas), while others project to the ventral striatum (in primates, for example, orbitofrontal, prefrontal and anterior cingulate cortices). A common view is that basal ganglia loops are anatomically and functionally segregated forming multiple *parallel circuits*, as emphasized originally by Alexander *et al*. [43]. However, several research groups have described ample anatomical substrates for interactions between circuits, such that multiple opportunities for crossover between streams exist [46–50]. We will develop the theme of intercommunication between basal ganglia loops considerably below, as it plays an important role in the intermixing and integration of brain signals that we suggest blur potential mental categories that can be instantiated by the vertebrate neuroarchitecture.

## 4. Amygdala

A central point of the present paper is that standard mental categories are ill-suited to investigating the brain basis of behaviour. Here, we describe how the amygdala participates in cortical–subcortical (technically, pallial–subpallial) loops that interlink a very wide spectrum of signals in a way that breaks down the barriers between purported mental domains.

In broad terms, the amygdala of mammals consists of (i) a basolateral complex (including lateral, basal and accessory basal nuclei and some cortical areas), and (ii) an *extended amygdala* (including the central nucleus, the medial nucleus and the complex of the BST [44,51]). In mammals, we refer to the amygdala as a subcortical structure (with the exception of a few cortical parts; for instance, involved in olfactory processing). Technically, based on the embryonic development of the telencephalon, the basolateral amygdala is mostly of pallial origin and the extended amygdala is mostly of subpallial origin [35,52]. In other words, as the brain develops, the embryonic division that originates the cortex also generates the basolateral amygdala, and the embryonic division that originates the basal ganglia also produces the extended amygdala.^7^

The embryonic origin and the regulatory genes expressed in each division during development explain the large population of glutamatergic neurons and the typical excitatory projections of the pallial part of the amygdala, as well as the considerable quantity of GABAergic neurons and the inhibitory projections that characterize the subpallial amygdala (reviewed by Medina *et al*. [35]). More recently, it was shown that part of the medial extended amygdala, previously thought to be subpallial, originates in a new division of the telencephalon, interposed between the subpallium and the hypothalamus, explaining the presence of abundant glutamatergic projection neurons in this region [55].

### (a) The pallial amygdala system

First, let us consider the anatomical pathways of the pallial amygdala with other parts of the pallium [56]. It has connections to frontal, parietal, cingulate, prefrontal, insular (both granular and agranular), temporal, olfactory and hippocampal cortices. Swanson & Petrovich [57] suggested naming this sector as the *fronto-temporal* amygdala due to its extensive interconnectivity with the cortex. Although this designation emphasizes the pathways with these parts of the cortex, it does not convey the fact that the pallial amygdala has extensive connectivity with *all* four pallial sectors discussed previously. Some examples in mammals (based on primates and rodents) are summarized next [58]. (i) Dorsal pallium: projections from lateral prefrontal cortical areas (Brodmann areas 8, 45, 46, parts of 9 and 12), with heavier connections originating from more caudal regions; projections from the amygdala to those areas are fairly light. (ii) Lateral pallium: major connections with the orbitofrontal cortex (especially the caudal aspect). (iii) Medial pallium: extensive connections with the hippocampal complex (fields CA3, CA2, CA1 and dentate gyrus; enthorhinal cortex and subiculum). Projections to the hippocampus are substantially stronger than input from the hippocampus. (iv) Ventral pallium: basolateral amygdala nuclei are richly interconnected (e.g. pathways between the lateral and the basal nuclei), as well as interconnected with the piriform cortex.

Given the extensive connectivity of the pallial amygdala with all sectors of the pallium, we propose that this area is not devoted to a single function but participates in a broad array of functions across the spectrum of traditional mental domains—emotion, cognition, action. Far from a ‘danger detector’ or a ‘fear centre’, the pallial amygdala is a hub that participates across multiple cerebral networks supporting diverse functions.

How is the pallial amygdala organized in other vertebrates? Given the emphasis in the literature on the survival-related functions of the amygdala (here both the basolateral and the extended amygdala), one would expect that the amygdala would be highly conserved and the task of identifying it across vertebrates would be relatively straightforward. However, this is far from being the case, and particularly problematic for the pallial amygdala. Despite the challenges, pallial amygdala-like regions have been identified across vertebrates (for discussion of some of the disputes in the literature, see Medina *et al*. [35] and Pessoa *et al*. [12]).

In birds, part of the proposed avian pallial amygdala-like region, the *caudal nidopallium*, is richly interconnected with all four sectors of the pallium. The reciprocal connectivity with other pallial areas is so extensive that the caudolateral nidopallium is considered functionally analogous (that is, functionally similar but not homologous) to the prefrontal cortex of mammals [59]. Instead, we suggest that the caudolateral nidopallium in birds is functionally similar to the pallial amygdala of mammals, which also shares a similar set of connections with different pallial sectors [12]. In other words, the extensive connectivity of the avian caudal nidopallium that has led some investigators to propose that it is functionally analogous to the mammalian frontal cortex is consistent with the extensive connectivity of the mammalian basolateral amygdala. The pallial amygdala is one of the most associative and integrative brain regions, especially in non-mammals.

The connectional systems of the pallial amygdala-like area of birds and non-avian reptiles (henceforth, referred to as ‘reptiles’) span multiple levels of the neuroaxis, allowing it to be involved in multifaceted signalling (related to external and internal realms). It exhibits exuberant connections with other pallial areas (as in mammals) and additional circuits via the basal ganglia, hypothalamus, subpallial extended amygdala and thalamus offer the potential for further signal communication, especially in birds where thalamic projections reach a broad spectrum of pallial areas (see the next section for elaboration of this point). Thus, the pallial amygdala in all amniotes, notably in mammals, birds and reptiles, is in a pivotal position for integrating multiple signals and participating in multiple functions that support effective behaviours in complex and dynamic ecological niches.

A pallial amygdala-like area has been identified in the ventral pallium of amphibians and teleost fishes [35,36,60], where several aspects of the connectivity are reminiscent of the amniote organization, including connectivity with other pallial regions [36]. For example, in frogs, the pallial amygdala (called the lateral amygdala) is reciprocally connected with several pallial areas including the rostral pallium, the lateral pallium and the olfactory bulb [61] and projects to the central amygdala and the BST, and then to the hypothalamus [36,62].

## 5. The telencephalic system involving the subpallial amygdala

**Figure 2.**
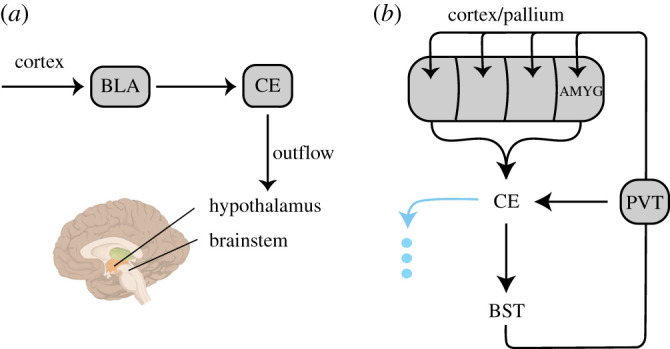
Amygdala circuits. (*a*) Outflow view of amygdala pathways, where information flows from the basolateral amygdala (BLA) to the central amygdala (CE), and then to regions important for autonomic and endocrine processing. (*b*) Basal-ganglia-type circuits involving the extended amygdala (CE and bed nucleus of the stria terminalus (BST)). A substantial input to the central amygdala originates from the basolateral amygdala, but all pallial sectors are involved to some extent. The arrow in cyan pointing away from CE represents the ‘outflow’ arrow of part (*a*). The loop through thalamus involves the paraventricular nucleus (PVT). (Online version in colour.)

Let us turn now to the subpallial amygdala, which in mammals includes the central amygdala. The central amygdala interfaces with the hypothalamus and brainstem in a manner that makes it an important component of endocrine and autonomic reactions to motivationally significant information [63]. The well-established *outflow* role of the central amygdala is frequently the target of experimental investigation (figure 2*a*). Here, we discuss a key component of the connectivity of the subpallial amygdala that places it within the context of cortical–subcortical circuits. In this view, the central amygdala plays a role comparable to that of the striatum in basal ganglia loops [12,44,64,65]. The importance of this perspective is that it extends the functional role of the central amygdala beyond autonomic and endocrine processes, bringing it to bear upon a broad array of processes that are not confined to those traditionally described as ‘emotion’ or ‘affective’ processing.

Researchers have noted that the central amygdala contains striatum-like GABAergic projection neurons, as well as other properties of striatal neurons (e.g. [44,57,66]). In addition, they share their origin in the striatal embryonic division [35,64]. Accordingly, it has been proposed that the central amygdala should be conceptualized as part of the striatum. As described next, the connectional logic of the central amygdala parallels that of basal ganglia loops in important ways (figure 2*b*).

The central amygdala receives inputs from the four major pallial compartments. In particular, inputs from the pallial amygdala (part of the ventral pallium) are extensive. The pallial amygdala interfaces with the extended amygdala much like the isocortex (that is, six-layered cortex) interfaces with standard basal ganglia loops (functionally, this also matches the integrative properties of the pallial amygdala, which receives massive inputs from across the cortex). The central amygdala projects to the BST (mostly its lateral part), which can be considered a pallidum-like region in terms of its molecular profile and embryonic origin ([67]; reviewed by the authors in [35,68]). The BST subsequently projects to the thalamus, which in turn projects to several pallial/cortical targets. The pathways from the BST to the thalamus target the paraventricular thalamic nucleus (PVT) and other midline nuclei [69,70].^8^ In all, the overall arrangement establishes a pathway through the central extended amygdala and back to the pallium [71].

Although views of the organization of classical basal ganglia loops are evolving and suggest a more open loop arrangement as opposed to strictly parallel streams ([46–50]; see also the next section), those through the extended amygdala clearly should be conceptualized as fairly open loops. In particular, the inter-pallial connectivity of the basolateral amygdala demonstrates the extensive influence of the extended amygdala loop on pallial function.^9^

## 6. Linking cortical–subcortical connectional systems

The organization of cortical–subcortical loops via the striatum constitutes a major large-scale organizational principle of the brain. Whereas basal ganglia loops involving different parts of the cortex were considered originally fairly segregated, recent evidence indicates a considerable amount of cross-talk between them. Understanding their intercommunication is important because it provides potential avenues to investigate interactions between multiple classes of signals—‘cognitive’, ‘affective’, ‘motor’.

**Figure 3.**
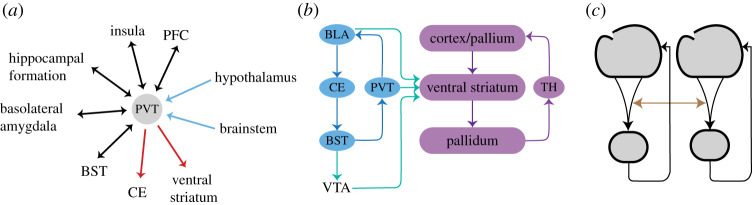
Interlinking of basal ganglia-type loops. (*a*) The paraventricular nucleus of the thalamus (PVT) functions as a hub region given its extensive interconnectivity. (*b*) The extended amygdala basal ganglia loop engages the PVT. This region has a considerable projection to the ventral striatum, therefore, linking the extended amygdala and ventral striatum basal ganglia loops. The PVT is also richly interconnected with multiple brain regions (see text). (*c*) Schematic of interlinked basal ganglia-type loops emphasizing that their integrative properties complement their organization in terms of separate loops. BLA, basolateral amygdala; BST, bed nucleus of the stria terminalis; CE, central amygdala; PFC, prefrontal cortex; PVT, paraventricular nucleus of the thalamus; TH, thalamus; VTA, ventral tegmental area. (Online version in colour.)

An important mode of information exchange between loops occurs via *hub regions*, namely, regions with fairly extensive connectivity. One such region is the PVT (figure 3*a*) (for a review of its connectivity, see [71,74,75]). In the extended amygdala system, the pathways from the BST to the thalamus target the PVT. The PVT also projects to the nucleus accumbens, effectively interlinking the extended amygdala and the ventral striatal connectional systems (figure 3*a*). The projections of the PVT have a remarkable property. Whereas the majority of neurons in the PVT project to the accumbens, most of them give off collaterals that innervate multiple subcortical targets, including the BST and central amygdala [74–78]. In other words, the same PVT neuron impacts responses across multiple target structures.

The functional relevance of this organization can be appreciated further by considering additional pathways. The PVT is reciprocally connected with pallial areas, such as the insular cortex, the prefrontal cortex (including orbitofrontal cortex), the hippocampal formation and the basolateral amygdala. All of these pallial sectors are themselves reciprocally interconnected, and project to the central extended amygdala and nucleus accumbens. In addition, the PVT receives substantial inputs from the hypothalamus and the brainstem. Together, the PVT is a key node for the interchange of affective and reward-relevant information and for modulating behaviour in a context-dependent manner. Indeed, recent research is uncovering its critical contributions during both appetitive and aversive processes [79–83].

Figure 3 illustrates another important channel for intercommunication between cortical–subcortical loops involving the ventral tegmental area (VTA). The BST projects to the VTA, a midbrain dopaminergic centre that projects to both the ventral striatum (nucleus accumbens) and the extended amygdala (including the central amygdala). The VTA also plays an important role in influencing the pallium, in particular, the basolateral amygdala and the prefrontal cortex. Taken together, the VTA occupies a pivotal position for interlinking cortical–subcortical loop-like systems.

Additional opportunities for cross-talk exist. Multiple pathways link pallial areas from different compartments, combining signals across basal ganglia loops at the *level of the pallium*. For example, in mammals, the pallial amygdala is reciprocally connected with the isocortex, the lateral entorhinal cortex and the hippocampal cortex [84–86]. The interconnectivity at the level of the pallium is not only a property of the mammalian brain but is present in birds and reptiles, too. For example, as discussed previously, the caudal nidopallium in birds and the equivalent area in reptiles (posterior dorsal ventricular ridge) is an area of the ventral pallium that is linked to all other pallial sectors. In particular, in birds, this area is reciprocally connected with the (i) dorsolateral pallium, which includes an entorhinal-like area as well as an orbitofrontal-like area [31,35,73]; (ii) the Wulst, a dorsal pallial region homologous to the isocortex in mammals; and (iii) the hippocampal formation in the medial pallium. In addition, as in mammals, this area in the ventral pallium of birds and reptiles projects to several areas of the subpallium of the forebrain, including the nucleus accumbens in the ventral striatum, the dorsal striatum and the subpallial amygdala [35,73] (figure 3*b*).

Together, the pathways discussed interlink dorsal and ventral basal ganglia loops, which are typically considered to be largely parallel/independent, in ways that are not usually considered (see also [45,53]). Importantly, because this organization is found not only in birds and mammals, but also in reptiles, this feature was likely present in the most recent amniote ancestor. The ample cross-talk between loops also suggests a major role of both the basal ganglia (both dorsal and ventral components) in processing non-motor signals, namely, those more closely aligned with contextual, aversive and appetitive signals. In the end, whereas it is important to understand some of the dominant roles of specific cortical–subcortical basal ganglia loops, it is equally important to understand the many ways in which they are coupled (figure 3*c*).

## 7. Mental categories and the vertebrate neuroarchitecture

Let us return to the question of mental categories studied in neuroscience. Are standard terms like ‘attention’, ‘memory’ and ‘decision-making’ useful for studying and describing the relationship between brain and behaviour? More directly, what should the neuroscientist care about? We argue that a comparative understanding of the general vertebrate neuroarchitecture strongly constrains the classes of mental processes in vertebrates. In particular, the functions supported by the neuroarchitecture do not align themselves well with the standard decomposition. In other words, in part, our argument is that the standard decomposition would require an organization that is relatively modular. We argue, instead, that fundamental principles of the neuroarchitecture indicate that it is *not*.

In particular, the neuroarchitecture is not ‘additive’—in the sense that new components are added atop an ancestral organization—as proposed by Edinger. As an example of non-additive changes, consider the organization of the basal ganglia. Whereas important components of its architecture are conserved across vertebrates, substantial differences are observed, too. In amphibians and reptiles, prominent pathways link the basal ganglia with the optic tectum [87–89], while a less extensive system interlinks the basal ganglia and the pallium.^10^ In reptiles and amphibians, basal ganglia loops involving the thalamus course through the *ventral* striatum, but an important addition is observed in birds and mammals, both of which include additional loops via the *dorsal* striatum (caudate-putamen). The considerable development and elaboration of connectional systems involving the cortex/pallium and basal ganglia can be viewed as reflecting, in part, the expansion of the thalamus and pallium in birds and mammals [90,91]. Taken together, the novel features of the bird and mammalian brain are not only related to the expansion and the increase in complexity of certain territories, but to the reorganization of existing circuits (e.g. a shift away from circuits involving the optic tectum), as new ones emerge (e.g. dorsal basal ganglia pathways through the thalamus).^11^

We conclude that the architecture of the brain is radically distinct from the one that would support circumscribed mental functions. Distributed brain circuits help solve challenging behavioural problems, and our claim is that interactions and integration at different levels are integral to that ability. In doing so, any purported standard mental property (such as ‘decision-making’) is, of necessity, deeply intertwined with others (such as ‘affective processing’).

## 8. Attention

We now turn to a discussion of a specific mental function, ‘attention’, and briefly illustrate the difficulties of mapping standard mental terms to the brain. Several investigators have pointed out that ‘attention’ is not a coherent concept, as it is linked to multiple processes ([9], and references therein). In fact, it is not even clear if attention is ‘cause’ or ‘consequence’. For example, Krauzlis *et al*. [54] argue that attention arises as a byproduct of circuits centred on the basal ganglia involved in value-based decision-making; in their view attention is an effect, not a cause. If one accepts the notion that attention is not a unified concept, how should it be conceptualized? As a first step, we propose to conceptualize it in terms of multiple *attention-like selection mechanisms*.

The advantage of doing so is that *selection* can be applied across multiple mental domains, including those that are conventionally described as motivational, affective, cognitive and so on. Doing so allows us to conceptualize the underlying processes as inherently *cutting across* domains and not, say, in terms of a ‘cognitive’ function, as typically done.

As an illustration, consider *affective attention*. Affectively significant visual items, such as those previously paired with shock, are behaviourally prioritized and detected faster [92–95]. Thus, they compete with other items more effectively during demanding conditions. Noting that the amygdala is involved in the processing of affectively significant information and that pathways from the basolateral amygdala reach nearly all levels of the ventral visual system (including primary visual cortex; [51]), researchers have suggested that such projections provide boosting signals to visual cortex when visual items are negatively valenced. Although this mechanism is frequently highlighted as the key one supporting the enhanced processing of emotion-laden visual items, several other mechanisms are likely involved [93]. For example, interactions involving the pulvinar nucleus of the thalamus and the basolateral amygdala likely support the behavioural advantage of negatively valenced visual items [96].^12^ More generally, we propose that both the pallial and the subpallial amygdala should be considered as important structures for selective attention-like processes, too [93,98]. For example, interactions between the basolateral amygdala and frontal and parietal brain regions (possibly involving indirect pathways) likely contribute to selection processes. Other circuits involve the subpallial extended amygdala. For example, projections from the central amygdala to the locus coeruleus can engage the latter area [99], which plays important roles in attention-like selection [100]. From an evolutionary perspective, it is noteworthy that multiple structures—including the optic tectum, thalamus and striatum—are also involved in *selection processes* closely tied to catching prey and avoiding predators [101]. Clearly, attention-like selection mechanisms are not confined to cortical circuits.

To conclude this section, we propose that it is fruitful to conceptualize ‘attention’ even more broadly than in terms of selection processes. Instead, it is useful to consider a broad family of ‘cooperative–competitive mechanisms’ that emerged philogenetically and that support gradually more sophisticated behaviours (Cisek [10] makes a related point in the context of ‘decision-making’). Such conceptualization encompasses, for example, circuits involving the extended amygdala and parabrachial nucleus that are relevant for the integration of threat information and feeding behaviour [102].

Cooperative–competitive mechanisms support a wide range of behaviours, typically combining diverse sources of evidence, including those related to the body and the external environment. This conceptualization helps shift the focus from ‘understanding attention’, say, to studying how particular brain circuits support particular types of behaviour.

## 9. Threat assessment

If the standard approach of relating functions and brain mechanisms is problematic, how should we proceed? We propose addressing the following question: What neural circuits/systems subserve specific classes of *behaviour*? Animals are confronted with environmental problems that must be solved to ensure reproductive success [103]. The focus on behaviour, especially in terms of the problems it has presumably evolved to solve, is of course the cornerstone of the *behavioural ecological* approach inspired by *ethology* [104,105]. As pointedly summarized by Fultot *et al.* [106]: the organism is viewed as a ‘seeker of stimulation rather than that of a processor of it’. Thus, to elucidate families of brain processes requires situating them in the context of ‘complex naturalistic behaviour’ (for a recent multi-author discussion, see [107]).

Consider the *threat imminence* framework, which proposes that, from the standpoint of an animal subject to predation, natural defensive processing should be understood in terms of three key stages [103]: pre-encounter, post-encounter and circa-strike. During pre-encounter, the animal's behaviour is constrained by the assessment of the probability of encountering a predator. During post-encounter, behaviour generally shifts markedly; animals frequently suppress behaviour, taking stock of the situation. Circa-strike behaviours may involve flight (if possible) or fight (usually as a last resort). Several related frameworks have been described, including the ethological approach by Blanchard and co-workers [108,109], defensive approach/avoidance systems [110] and the extension of the predator imminence model to humans by Mobbs and collaborators [111].

Ethologically inspired work has a different flavour compared to the standard neuroscience approach. Research themes include foraging, parental care, predator–prey interactions, sexual selection and social behaviours. Whereas these topics overlap rather little with those motivated by cognitive psychology, other lists of ecological themes are probably more familiar to a wider group of neuroscientists: signal detection, signal localization, memory acquisition, storage and recall, motivation, coordination and top–down control [112]. Indeed, some investigators have proposed combining ethological approaches with traditional systems neuroscience, in particular by studying complex behaviours in more natural conditions while recording movement, performing temporally specific perturbations and recording from large numbers of neurons during freely moving behaviours [107].

**Figure 4.**
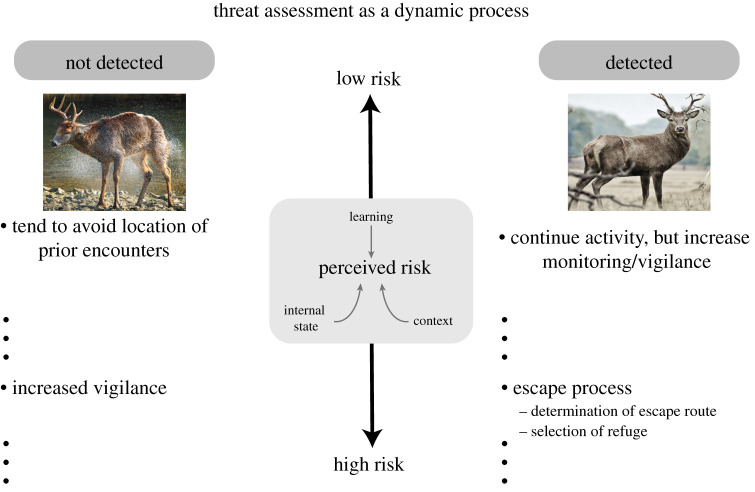
Threat assessment as a dynamic process. (Online version in colour.)

Let us consider the processes of *threat assessment* during predator–prey interactions. Far from stereotyped, such processes can be highly complex and flexible in naturalistic conditions [113] (figure 4). The entire process is dynamic such that behaviours are continuously adjusted based on a large set of interacting variables involving both prey and predator while they navigate their mutual environment. Heuristically, we can refer to a level of *risk* that is continuously monitored and updated, and which is context-dependent and based on prior experience (see [114,115]). For example, certain patches of a habitat may be associated with previous encounters that were more dangerous [116]. At the broadest level, we can refer to a threat as *detected* or *not detected*. When threat is not detected, risk assessment might dictate avoiding locations of prior predator encounter, as well as adjusting vigilance levels depending on available cues. As the animal navigates locations of increased risk, they may avoid the territory altogether, but this choice could be overridden by factors such as high levels of thirst or hunger.

When threat is detected, ensuing escape responses would be expected. However, animals are not simple stimulus–response devices. If risk is low, prey will continue ongoing activity, but channel it in specific directions; for example, continue grazing but in a manner that at least maintains the distance to the predator. When risk is higher, escape-related behaviours will ensue but with *vigour* that is commensurate with the condition at hand. For example, if the risk is moderate, the animal might simply increase its distance from the predator. More generally, the escape process is informed by multiple internal (e.g. hunger, fatigue, bodily health and sexual arousal) and external (e.g. distance and predator behaviour) variables. In particular, escape involves the determination of an adequate route that is tightly coupled with the selection of appropriate refuge. This selection accounts for the safety value of the shelter, the distance and position of the predator relative to the shelter and potential competition for access (e.g. the burrow is frequently occupied by other animals). For further discussion, see Evans *et al*. [113] and Branco & Redgrave [117].

Ultimately, the term ‘threat assessment’ can be used as a shorthand for a series of interrelated processes that determine an animal's behaviour in the context of potential and actual encounters with predators. As such, the study of the neural basis of threat assessment must be undertaken in naturalistic settings that approximate the range of behaviours observed in nature, coupled with rich characterization of behaviours of multiple interacting actors, including predators. How is such ecological outlook related to traditional systems neuroscience approaches [118–120]?

In the preceding discussion of threat assessment, we can identify several instances in which terms like ‘attention’, ‘emotion’ or ‘decision-making’ could possibly be used descriptively. For example, as an animal navigates its environment and perceived risk increases to moderate levels, it will ‘pay more attention’ to certain aspects of the environment. Cues associated with the presence of predators will gain increased salience. Many of these cues will be laden with affective significance from past encounters, and will engage circuits that are typically described with labels such as ‘emotion processing’ or involving ‘attention–emotion’ interactions. Furthermore, the presence of the predator will invoke a series ‘attentional’ processes related to the acquisition and selection of sensory information, and will be associated with head turning or body movements.^13^

By and large, the standard neuroscience approach attempts to compartmentalize and isolate behaviours, such as when studying eye/head or ‘attentional movements' to salient visual items. But, in general, behavioural ‘decision-making’ involves a complex interplay of multiple variables that collectively contribute to action choices. As an illustration, consider the following type of decision by a prey: because (i) the hunger level of the animal is high, (ii) a relatively unimpeded route to safety is possible, (iii) the predator is alone, (iv) the predator is not currently approaching, and (v) this type of predator (cheetah, say) has only been more dangerous when attacking the animal from stealth, then the animal may continue grazing. Whereas some of the variables above are temporally stable (past history of encounter with the animal), others may fluctuate temporally (e.g. whether the predator is alone or not). Because behavioural processes build upon a very large set of dynamic variables, whereas the standard account (e.g. ‘attention mechanisms', ‘decision-making’) provides a potential heuristic description, the latter fails to capture the rich interdependence of the multiple mechanisms that support behaviours. In the end, the standard account provides a language that emphasizes independence and separation, where a language of *interaction* and *integration* is needed.

To conclude, we propose that threat assessment should be viewed as a highly *dynamic process*. Whereas actions must per force occur sequentially—escape initiation → escape execution → escape termination [122]—we suggest that it is necessary to conceptualize threat assessment in a continuous fashion. In this manner, as some mechanisms and processes are engaged, they lead to actions that alter environmental relationships, which in turn are continuously assessed to guide further actions.

### (a) Large-scale circuits and mental functions

What are the neural circuits involved in dynamic threat assessment? We suggest that the overall process cannot be subdivided into separate systems that are engaged during pre-encounter defence versus post-encounter defence, for example. More generally, there is no single underlying system for threat assessment. But this does not mean that we cannot tackle the problem of the neural basis of behaviour. In other words, whereas the standard mental domains do not provide an adequate framework, it is still possible to study the *coupling* between dynamically engaged distributed neural circuits and complex, dynamic behaviours. By ‘coupling’, we mean the set of *regularities* between brain and behaviour, in particular how *variability* in behaviour is linked to *variability* in neural circuits.^14^ Importantly, this mapping is not one-to-one (one behaviour, one circuit), but *many-to-many* (one behaviour can be linked to multiple circuit instantiations, and one circuit can be linked to multiple behaviours) (see [124]).^15^

**Figure 5.**
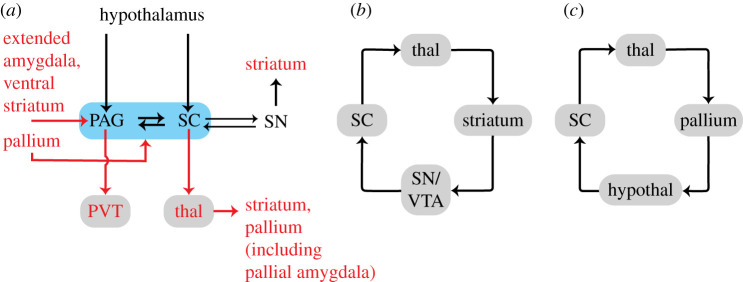
Large-scale circuits that participate in threat assessment. (*a*) Superior colliculus–periaqueductal grey circuit (see dashed outline). Parts in red mark some explicit bridges to/from the circuits discussed in figures 2 and 3, including the PVT, which illustrates the important role of connector hub regions. (*b*) The superior colliculus is part of subcortical loops. (*c*) The superior colliculus is part of loops with the pallium. Hypothal, hypothalamus; PAG, periaqueductal grey; PVT, paraventricular nucleus of the thalamus; SC, superior colliculus; SN, substantia nigra; thal, thalamus; VTA, ventral tegmental area. (Online version in colour.)

Although relatively little is known in mammals, as a starting point, we suggest that it is useful to anchor threat assessment circuits on the *superior colliculus* and *periaqueductal grey* (PAG) (figure 5*a*). The superior colliculus is often emphasized as a fairly direct sensorimotor interface, but has extensive anatomical connectivity throughout the brain, including extensive visual inputs and outputs to areas regulating head orientation and gaze direction [125]. Several investigators have noted its participation in defensive behaviours (for reviews, see [117,126–128]; for evidence in primates, see [129–131]), in addition to well-known involvement in target selection and related functions commonly described as ‘attentional’ [54,101,132]. The superior colliculus works in close connection with the PAG (they are bidirectionally connected), and the deep layers of the former may form an integrated anato-functional unit with the latter [133], a region heavily involved in defensive behaviours ([134]; see also [135]). The superior colliculus and the PAG receive inputs from the hypothalamus, too, so *bodily context* and other *state-related* signals can be taken into account.

The local circuits between the superior colliculus and the PAG do not work in isolation, and have the ability to influence behaviours in several ways, for example, via dopaminergic signalling involving the VTA and the substantia nigra (figure 5*a*). Importantly, the superior colliculus has the potential to become embedded into large-scale circuits in several ways. The superior colliculus is part of loops with the subcortex via the thalamus and the basal ganglia (for review, see [136]) (figure 5*b*). Additionally, via the thalamus, the superior colliculus is linked to the pallium (including the pallial amygdala), which also influences the superior colliculus/PAG via the hypothalamus (figure 5*c*).

More generally, and critically, the PAG–superior colliculus circuit readily engages with the large-scale connectional systems discussed previously (figures 2 and 3), for example, via the thalamus, striatum and pallium (see parts in red of figure 5*a* and *b*,*c*). For example, both the superior colliculus and the PAG receive inputs from the pallium, the hypothalamus and some midbrain tegmental areas (such as the VTA); these territories, in turn, receive basal ganglia and amygdala inputs, among others. The integrative potential of the PAG–superior colliculus circuit is, therefore, enormous and, via ascending and descending projections, the circuit is involved in a wealth of behaviours.

In the context of threat assessment, the neural circuits engaged (in a species-specific fashion) combine a large number of internal (state of the animal) and external variables (e.g. is there a path to safety?), with prior learning and future-oriented scenario simulations in a situation- and context-dependent fashion (e.g. how much time does the animal have?). In this manner, the scale of the circuit engaged is temporally and condition-specific, ranging from more circumscribed interactions involving fewer brain regions and territories to large-scale circuits across the neuroaxis that span a substantial amount of the brain. Thus, one cannot point to the brain and say ‘here's where threat assessment happens'. Instead, it is an outcome of the distributed and potentially large-scale mechanisms that support behaviour at a specific point in time, and how the brain–behaviour coupling evolves temporally.

Returning to standard mental functions (perception, attention, cognition, etc.), can they be used to describe subcomponents of threat assessment? Whereas it is conceivable to use them while taking into account the considerations raised throughout this piece, it would require a major shift from key ways in which they are used by neuroscientists. What is more, in typical usage, they define a research agenda that, in many ways, is ‘reversed’. For example, a considerable amount of energy has been devoted by neuroscientists to uncover ‘the emotional brain’, an endeavour that we view as futile given that the vertebrate brain does not conform to the boundaries of mental domains.

## 10. Back to the brain and mental functions

What kind of system is the brain? The brain has evolved to provide adaptive responses (‘functional responses' in the evolutionary sense) to the problems that living beings face in order to survive and reproduce. Our brief discussion of the vertebrate brain focused on principles of the organization of cortical–subcortical loop-like circuits, as well as forebrain–midbrain interactions. A key goal was to illustrate how the neuroarchitecture supports *combinatorial* brain connectivity—from region A to region B via multiple routes. Functionally, circuits form *dynamically* such that specific populations of neurons across areas coalesce into coherent functional units. The overall organization is *heterarchical*, namely without fixed hierarchies.^16^

The neuroarchitecture of vertebrates involves long-range circuits that span the midbrain, thalamus and pallium/cortex, among other regions. We suggest that the anatomy supports a high degree of behavioural flexibility, allowing animals to cope with the multifaceted interactions they engage in involving predators, prey, potential mates and so on. In species with more malleable behaviours, behavioural success benefits from circuits that can form *flexibly*, as the number of conditions related to the internal and external worlds of the animal are exceedingly high.

As neural circuits support behavioural elements, we suggest that the level of *behaviour* provides the appropriate language for considering the mapping between the brain and mind. The mental domains of the neuroscience vocabulary (attention, cognitive control, etc.), with their origins detached from the study of animal behaviour, provide problematic conceptual anchors. From the present perspective, the conclusion by several authors that categories of mental terms are too heterogeneous to be conceptually useful is thus unsurprising. Furthermore, based on the present framework, there is no confusion between the phenomenon to be explained and the mechanism used to explain it; for example, in using ‘attention’ to refer to phenomena that engage ‘attentional mechanisms' [9]. The framework also protects against mixing cause and effect; e.g. is attention a causal agent or a functional consequence of circuits with specific roles [54]?

In conclusion, we suggest that the vertebrate neuroarchitecture does not respect the boundaries of mental terms, and propose that situating research in terms of *complex, naturalistic behaviours* provides a more promising approach.

Ultimately, unravelling the complex dynamic mapping between brain and behaviour will require moving past notions of the mind that have dominated neuroscience for a century and a half.

## Footnotes

1 Quotes and references from this paragraph are from Anderson [2].

2 According to Merriam-Webster, perception is the awareness of the elements of environment through physical sensation; or, physical sensation interpreted in the light of experience.

3 Our treatment follows closely the one by Hommel *et al*. [9], which focused on *attention*.

4 Echoes of Edinger's ideas are of course evident in the work of Paul MacLean, who proposed the idea of the ‘triune brain’.

5 Some time ago, one of the authors (L.P.) was invited to write a chapter on the ‘emotional brain’ for a textbook on neuroscience with a clinical angle. For reference, L.P. inspected the chapter of a prior edition published in 2008. The chapter, entitled ‘The Limbic System’, based its content on the so-called circuit of Papez, a largely speculative proposal from 1937, which somewhat unbelievably is still influential despite being completely outdated.

6 Recently, new proposals of a ring-like organization of the pallium have emerged (for example, [32,33]). Although interesting, the proposals are largely cortico- and mammalian-centred, and less suited to explain the pallium organization in non-mammals. As such, ring-like models cannot yet be used to extract general organizing principles that apply to vertebrates more generally. Accordingly, in our discussion, we embrace a four/six pallial sector scheme.

7 The embryonic origin and the regulatory genes expressed in each division during development explain the large population of glutamatergic neurons and the typical excitatory projections of the pallial part of the amygdala, as well as the considerable quantity of GABAergic neurons and the inhibitory projections that characterize the subpallial amygdala (reviewed by Averbeck *et al*. [53]). More recently, it was shown that part of the medial extended amygdala, previously thought to be subpallial, originates in a new division of the telencephalon, interposed between the subpallium and the hypothalamus, explaining the presence of abundant glutamatergic projection neurons in this region [54].

8 The BST also projects to the hypothalamus, thalamus and brainstem [69].

9 This is even more evident when considering the *medial extended amygdala*, a nuclear complex originating from multiple embryonic neurons, some glutamatergic and others GABAergic [35,55]. The medial amygdala projects to the hypothalamus directly and by way of the medial BST. It also projects to the thalamus (PVT), to the ventral tegmental area (which has ample modulatory influence on the basal ganglia, amygdala and prefrontal cortex, among other areas) and to the periaqueductal grey [72]. Overall, the medial amygdala is part of social processing networks [73] involving the interactions between multiple functional systems.

10 In birds, prominent pathways link the basal ganglia with the optic tectum, too, but extensive connections between the basal ganglia and the pallium are present as well.

11 Basal ganglia pathways to the optic tectum continue to be important in birds, but new ones through the thalamus were added.

12 Midline thalamic nuclei, projecting to the pallial amygdala and prefrontal cortex, are involved too [97].

13 Perception and attention, therefore, take place in close coordination with—and in the context of—action [121].

14 For the important notion that behaviour can act as an enabling constraint on neural activity, see Raja & Anderson [123].

15 When we say ‘one behaviour can be linked to multiple circuit instantiations’, we mean mostly that the same behaviour can be instantiated by different, though possibly overlapping, neural circuits. More generally, this issue is complex and deserves further treatment, as even small ‘subregions’ are comprised by diverse and spatially overlapping neuronal populations, such as those identified by chemical/genetic markers.

16 At specific times, some regions can exert a stronger influence on others in a way that could be described in terms of hierarchical control, especially when ‘higher’ regions influence so-called lower regions. Unfortunately, such descriptions are almost always unhelpful and carry with them antiquated notions of brain organization.

## Data accessibility

This article has no additional data.
